# Tunicamycin enhances the antitumor activity of trastuzumab on breast cancer *in vitro* and *in vivo*

**DOI:** 10.18632/oncotarget.5334

**Published:** 2015-10-12

**Authors:** Xiqian Han, Xiaobing Zhang, Hui Li, Shengshi Huang, Shu Zhang, Fengshan Wang, Yikang Shi

**Affiliations:** ^1^ National Glycoengineering Research Center, School of Pharmaceutical Science, Shandong University, Jinan, China

**Keywords:** trastuzumab, tunicamycin, breast cancer

## Abstract

Trastuzumab, a humanized monoclonal antibody targeting HER2, has demonstrated clinical benefits for women with HER2-positive breast cancer; however, trastuzumab resistance remains the biggest clinical challenge. In this study, results showed that tunicamycin, an inhibitor of N-glycosylation, synergistically enhanced the antitumor activity of trastuzumab against HER2-overexpressing breast cancer cells through induction of cell cycle arrest and apoptosis. Combined treatment of tunicamycin with trastuzumab dramatically decreased the expression of EGFR family and its down signaling pathway in SKBR3 and MCF-7/HER2 cells. Tunicamycin dose-dependently inhibited tumor growth in both of SKBR3 xenografts and MCF-7/HER2 xenografts. Optimal tunicamycin without inducing ER stress in liver tissue significantly increased the antitumor effect of trastuzumab in MCF-7/HER2 xenografts. Combinations of trastuzumab with N-glycosylation inhibitors tunicamycin may be a promising approach for improving clinical efficacy of trastuzumab.

## INTRODUCTION

HER2 (also named as ErbB2) is a member of the EGFR receptor tyrosine kinase (RTK) family, which also include EGFR, HER3, and HER4. HER2 is activated by the formation of homodimers or heterodimers with other EGFR receptors. HER2 is generally regarded as the preferred heterodimerization partner for each of the ligand-bound EGFR receptors [1]. Two key signaling pathways activated by the EGFR family dimers are the MAPK pathway, which stimulates proliferation, and the PI3K/Akt pathway, which promotes tumour cell survival. The PI3K/Akt pathway is the predominant oncogenic pathway downstream of HER2 [2]. HER2 plays important roles in cell growth, survival, and differentiation in a complex manner. HER2 is overexpressed in 25–30% of human breast cancers and its overexpression is associated with more aggressive disease behavior and poor response to chemotherapy [3–5].

Trastuzumab is a monoclonal antibody that targets the HER2 extracellular domain, induces uncoupling of heterodimers, and inhibits downstream signaling. The mechanisms of the antitumor activity of trastuzumab mainly include prevention of HER2-receptor dimerization, increased endocytotic destruction of the receptor, inhibition of shedding of the extracellular domain, and activation of immune response through antibody-dependent cellularcytotoxicity (ADCC) and complement-dependent cytotoxicity (CDC) [6, 7].

Trastuzumab has significantly improved outcomes for patients with HER2- overexpressing breast cancer; however, innate and acquired trastuzumab resistance has increasingly occurred and remains the biggest clinical challenge. Many patients with HER2-overexpressing breast cancer either do not respond to initial therapy or develop acquired resistance to trastuzumab within one year. Because the HER2 signaling pathway is a complex biological network, inhibition of the HER2 oncogenic pathway with trastuzumab may result in compensatory crosstalk and activation of alternative signaling pathways, which contribute to the trastuzumab resistance. EGFR and HER3 overexpression might be responsible for acquired resistance to trastuzumab [8]. EGFR and HER3 expression is substantially increased after long-term trastuzumab exposure of breast cancer cell lines to trastuzumab [8]. Trastuzumab has been found to abrogate ligand-induced EGFR-HER2 binding and ligand-independent HER2-HER3 association; however, trastuzumab treatment has only a minor effect on ligand-induced HER2-HER3 dimerization [9, 10]. Activated PI3K/Akt pathway also induces strong trastuzumab resistance [11]. The knowledge of these mechanisms of trastuzumab resistance has driven the development of new drugs or drug combinations. Trastuzumab has widely been used clinically in combination with docetaxel, carboplatin or capecitabine in patients with HER2-overexpressing breast cancer and gastric cancer [12–14]. Combining trastuzumab with one or two chemotherapy drugs yields better response rate than single agent probably due to their complementary mechanisms of action. Therefore, a therapeutic strategy that disrupts signaling from multiple RTKs may have the potential advantage of blocking both the primary and the compensatory signaling mechanisms that have been shown to contribute to trastuzumab resistance.

N-linked glycosylation (NLG) is a highly regulated and critical step in the maturation of transmembrane RTK glycoproteins. Tunicamycin, a nucleoside antibiotic, inhibits the first step in the biosynthesis of N-linked oligosaccharides in cells. Consequently, the glycoproteins are not folded and cause an accumulation of mis- or unfolded glycoproteins in the Endoplasmic Reticulum (ER), resulting in the ER stress induction and apoptotic cell death [15, 16]. Previous reports have shown that disruption of N-linked glycosylation could reduce protein levels of RTK and downstream signaling pathway, suggesting that inhibition of NLG with tunicamycin is an alternative mechanistic approach to reduce both oncogenic signaling and the mechanisms of therapeutic resistance [17]. NLG inhibition produced marked radiosensitization in cancer cell lines but did not radiosensitize nontransformed cells [18]. Tunicamycin has been shown to enhance the susceptibility of lung cancer cells and sensitize resistant cell lines to Erlotinib [19]. Tunicamycin sensitizes human prostate cancer cells to TRAIL-induced apoptosis by increased expression of DR5 protein [20]. Tunicamycin also potentiate cisplatin-induced cytotoxicity in human cell lines [21]. Tunicamycin inhibits angiogenesis in nude mice by decreasing VEGF expression [22]. All these reports have confirmed that tunicamycin-induced disruption of NLG has a considerable advantage by both targeting multiple receptor types and sensitizing cancer cells to antitumor drugs.

Trastuzumab resistance pathways are multiple, interconnected and autonomous. Tunicamycin can inhibit N-glycosylated receptors and downstream signaling pathways. In this report, we test the antitumor activity of tunicamycin alone and in combination with trastuzumab on breast cancer cells with low or high HER2 expression. We also found out the optimal dose of tunicamycin which can inhibit N-glycosylation and decrease RTK expression without causing toxicity. We proposed that inhibition of the N-glycan biosynthesis by tunicamycin may be a promising therapeutic strategy for enhancing the sensitivity of cancer cells to trastuzumab.

## RESULTS

### Effect of tunicamycin and trastuzumab combination on the proliferation of breast cancer cells

**Figure 1:**
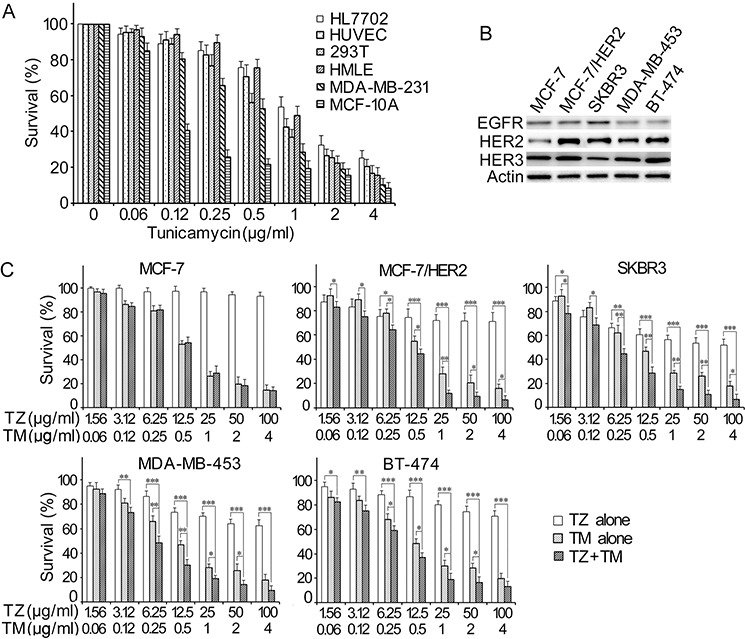
Growth inhibitory effects of tunicamycin alone and in combination with trastuzumab in breast cancer cell lines and in normal human cell lines The cells were exposed to either tunicamycin alone **A.** or in combination with trastuzumab **C.** for 96 h. **B.** The expression of EGFR family in breast cancer cells which were used in this study to evaluate the combined effects. The growth inhibitory effects were determined by SRB assay. The results were derived from three independent experiments performed in triplicate. TZ means trastuzumab, TM means tunicamycin. *P < 0.05, **P < 0.01, ***P < 0.001.

In order to investigate whether tunicamycin inhibit growth against tumor cell lines as the same manner as in normal cell lines, SRB assay was performed in several breast cancer cell lines as well as in some normal cell lines such as MCF-10A, HL7702, HEK293T, HMLE and HUVEC. As shown in Figure 1, exposure to tunicamycin for 96 h at various concentrations ranging from 0.125 μg/ml to 4 μg/ml resulted in a dose-dependent inhibition of cell growth in all tested cell lines. Tunicamycin-induced growth inhibition was not dependent on HER2 expression levels in breast cancer cells. The cytotoxicity produced by tunicamycin in breast cancer cells was similar to that in normal cells. Normal breast cells MCF-10A was most sensitive to tunicamycin; however, normal hepatocyte cells HL7702 was more resistant to it (Figure 1A). To investigate the effects of combined treatment of tunicamycin with trastuzumab on cell growth, we used low HER2-expressing breast cancer cells MCF-7 and HER2-overexpressing cells such as MCF-7/HER2, SKBR3, MDA-MB-453 and BT-474. The expression levels of EGFR family in these cell lines were shown in Figure 1B. In agreement with previous reports, trastuzumab inhibited proliferation of HER2-overexpressing MCF-7/HER2, SKBR3, MDA-MB-453 and BT-474 cells. In HER2-overexpressing cells, treatment with this drug combination resulted in a significant growth inhibitory effect when compared with the drug being added alone (Figure 1C). In MCF-7 cells, that were resistant to trastuzumab, combination treatments did not dramatically enhance the effect of the individual drug treatments (Figure 1C). In order to determine the enhanced effects are additive or synergistic, drug interaction was analyzed by the CalcuSyn Software (Biosoft, Cambridge, UK) and expressed as combination index (CI). As shown in Figure 1C and Table 1, the results indicated that tunicamycin and trastuzumab synergistically inhibited cell growth in HER2-overexpressing breast cancer cell lines.

**Table 1:** The combined effects of trastuzumab and tunicamycin on cell growth

Dose (μg/ml)		CI				
Trastuzumab	Tunicamycin	MCF-7	MCF-7/HER2	SKBR3	MDA-MB-453	BT474
1.56	0.0625	1.14	0.84	0.82	1.05	0.72
3.125	0.125	1.06	0.57	0.73	0.74	0.85
6.25	0.25	1.24	0.59	0.38	0.45	0.68
12.5	0.5	0.85	0.59	0.35	0.39	0.46
25	1	0.93	0.30	0.31	0.41	0.31
50	2	0.93	0.50	0.43	0.57	0.50
100	4	1.12	0.72	0.54	0.70	0.76

The cells were exposed to trastuzumab, tunicamycin, and both at the indicated concentrations for 96 h. The growth inhibitory effects were determined by RSB assay. CI was calculated by using CalcuSyn software. The results were derived from three independent experiments performed in triplicate.

### Effect of tunicamycin and trastuzumab combination on cell cycle

**Figure 2:**
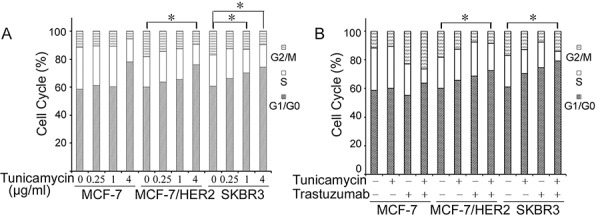
Effects of tunicamycin alone A. and in combination with trastuzumab B. on cell cycle distributions in breast cancer cell lines MCF-7, MCF-7/HER2 and SKBR3 The cells were treated with tunicamycin (1.0 μg/ml), trastuzumab (10 μg/ml), or combined drugs for 24 h and then analyzed for cell cycle distributions by flow cytometry. Representative experiments were carried out at least three times. *P < 0.05.

Flow cytometry analysis was performed after cancer cells were treated with different concentrations of individual or combination drugs for 24 h. Results showed that tunicamycin alone induced G0/G1 arrest with a dose-dependent manner in MCF-7, MCF-7/HER2 and SKBR3 cells (Figure 2A). As reported previously, trastuzumab also increased the population of cells at G0/G1 phase in MCF-7/HER2 and SKBR3 cells; however, trastuzumab did not induce cell cycle arrest in MCF-7 cells. Compared with individual treatment, the combined treatment of 1.0 μg/ml tunicamycin with 10 μg/ml trastuzumab dramatically enhanced the G0/G1 arrest in MCF-7/HER2 and SKBR3 cells, but not in MCF-7 cells (Figure 2B). Above results demonstrated that enhanced growth inhibitory effect of combined treatment in HER2-overexpressing cancer cells was partly due to the increased G0/G1 arrest.

### Effect of tunicamycin and trastuzumab combination on apoptosis

**Figure 3:**
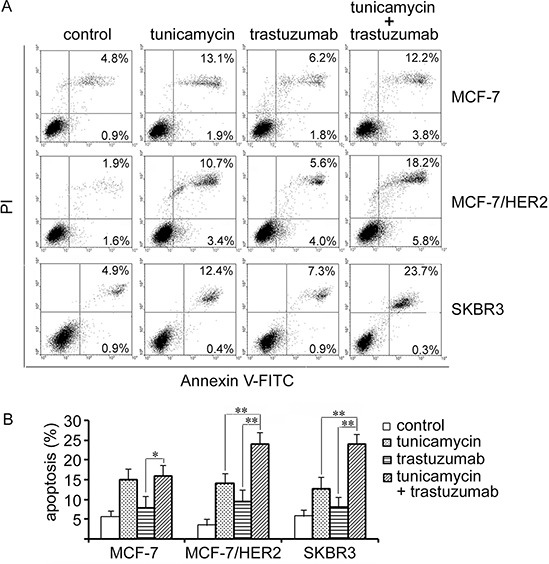
Effects of tunicamycin and trastuzumab treatment on apoptosis in breast cancer cell lines MCF-7, MCF-7/HER2 and SKBR3 The cells were treated with tunicamycin (1.0 μg/ml), trastuzumab (10 μg/ml), or a combination of both agents for 24 h and then analyzed for apoptosis. Representative experiments were carried out at least three times. *P < 0.05, **P < 0.01.

Annexin V-FITC/PI analysis was used to examine the percentage of apoptosis in MCF-7, MCF-7/HER2 and SKBR3 cells following treatment with tunicamycin, trastuzumab, or their combination for 24 h. Tunicamycin treatment alone induced apoptosis in a dose-dependent manner (data not shown). Trastuzumab treatment alone slightly improved apoptotic cells in MCF-7/HER2 and SKBR3 cells. As shown in Figure 3, the combination of tunicamycin (1.0 μg/ml) with trastuzumab (10 μg/ml) resulted in significant increases in the percentage of apoptotic cells as compared with either tunicamycin or trastuzumab treatment alone in MCF-7/HER2 and SKBR3 cells (Figure 3).

### Effect of tunicamycin and trastuzumab combination on cell signaling pathways

To investigate the mechanism responsible for the enhanced growth inhibition of the combination treatment of tunicamycin with trastuzumab, we investigated the signal transduction pathways correlated to EGFR family, ER stress, cell cycle and apoptosis.

**Figure 4:**
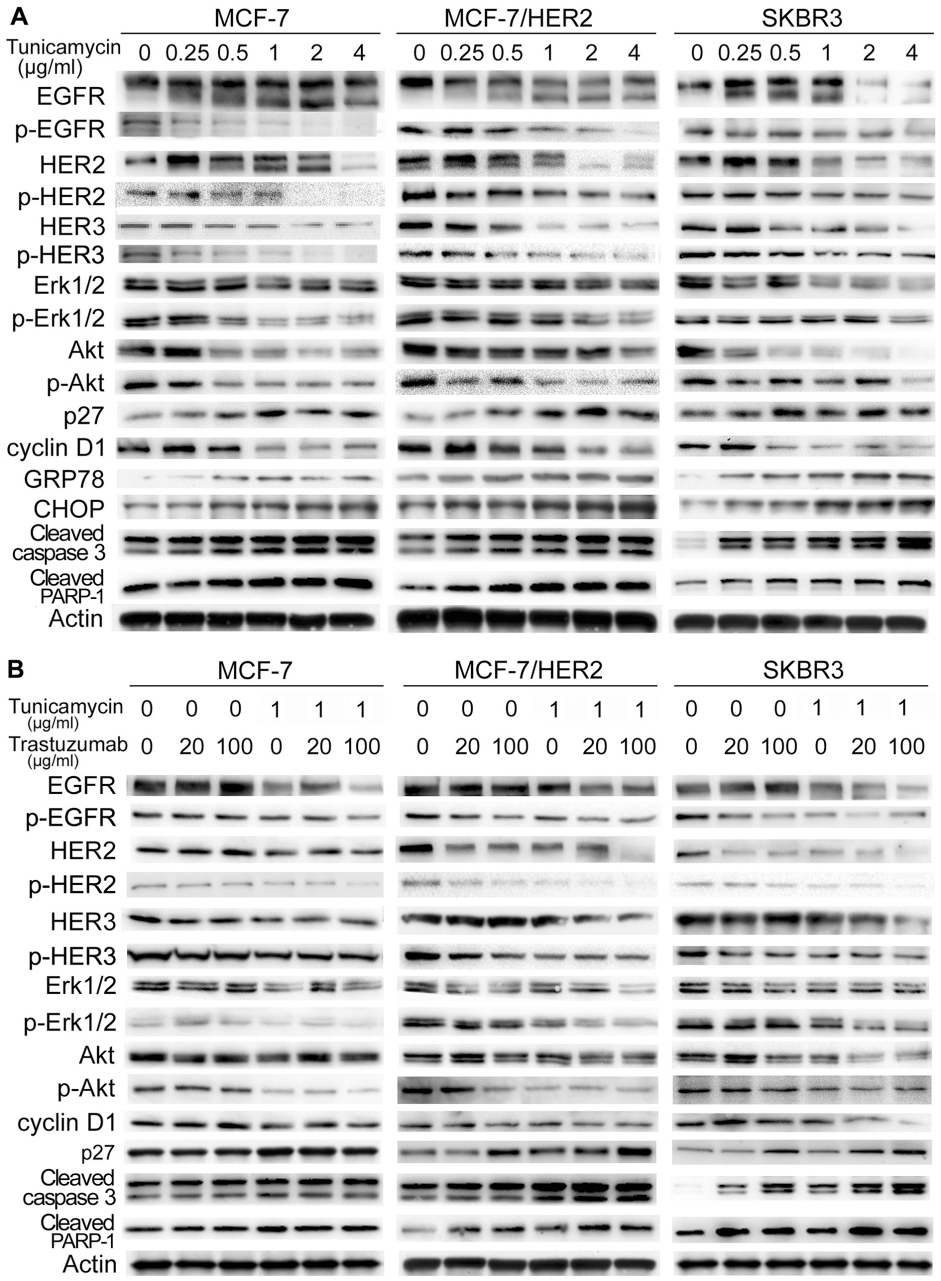
Effects of tunicamycin alone A. and in combination with trastuzumab B. on protein expression in breast cancer cell lines MCF-7, MCF-7/HER2 and SKBR3 The cells were treated with tunicamycin, trastuzumab, and the combination of these two drugs for 24 h and were then harvested for western blot analysis. Representative experiments were carried out three times.

Tunicamycin disrupted the protein expression and phosphorylation levels of EGFR, HER2 and HER3 in a dose-dependent manner in MCF-7, MCF-7/HER2 and SKBR3 cells. Tunicamycin treatment produced full sized EGFR and smaller molecular EGFR in three breast cancer cell lines. Similarly, full sized HER2 and smaller molecular HER2 were also observed after tunicamycin treatment with varying concentrations in breast cancer cells, indicating that tunicamycin induced unglycosylated EGFR and HER2. Tunicamycin-induced disruption of Erk1/2 and Akt were also accompanied with a decrease of their phosphorylation levels (Figure 4A). As previous reports, trastuzumab inhibited the HER2, Erk1/2 and Akt phosphorylation in MCF-7/HER2 and SKBR3 cells, not in MCF-7 cells (Figure 4B). As shown in Figure 4B, the fixed dose of tunicamycin at 1.0 μg/ml significantly enhanced trastuzumab-induced decreases of EGFR, HER2, HER3, Erk1/2 and Akt, as well as their phosphorylation levels in MCF-7/HER2 and SKBR3 cells, suggesting that MAPK and PI3K/Akt signaling pathways were greatly inhibited by the combination of tunicamycin and trastuzumab in HER2-overexpressing breast cancer cells.

We next examined the expression of cell-cycle regulators, such as cyclin D1 and cyclin dependent kinase inhibitor p27, which are essential for G1/S phase progression, in breast cancer cells treated with trastuzumab. Tunicamycin treatment resulted in concentration-dependent increase of p27 and decrease of cyclin D1 in all three breast cancer cells (Figure 4A). Trastuzumab alone also enhanced p27 expression with no affection on cyclin D1 expression in MCF-7/HER2 and SKBR3 cells. The protein level of p27 was significantly increased in response to the combination treatment of tunicamycin and trastuzumab in HER2-overexpressing cancer cells. The same changes were not found in MCF-7 cells with lower HER2-expression (Figure 4B). We examined whether low doses of tunicamycin with a little cytotoxicity would cause ER stress. As shown in Figure 4A, tunicamycin treatment alone obviously enhanced the expressions of CHOP and GRP78 in MCF-7, MCF-7/HER2 and SKBR3 cells. Above results showed that ER stress induced by tunicamycin may contribute to the growth inhibitory effects of tunicamycin. Tunicamycin dose-dependently increased the expression of activated caspase 3 and inactivated PARP in three cell lines; however, combined treatment obviously enhanced the expression of cleaved caspase 3 and cleaved PARP as compared with the treatment of either drug alone in MCF-7/HER2 and SKBR3 cells (Figure 4A and 4B).

### Effect of tunicamycin and trastuzumab combination on tumor growth *in vivo*

**Figure 5:**
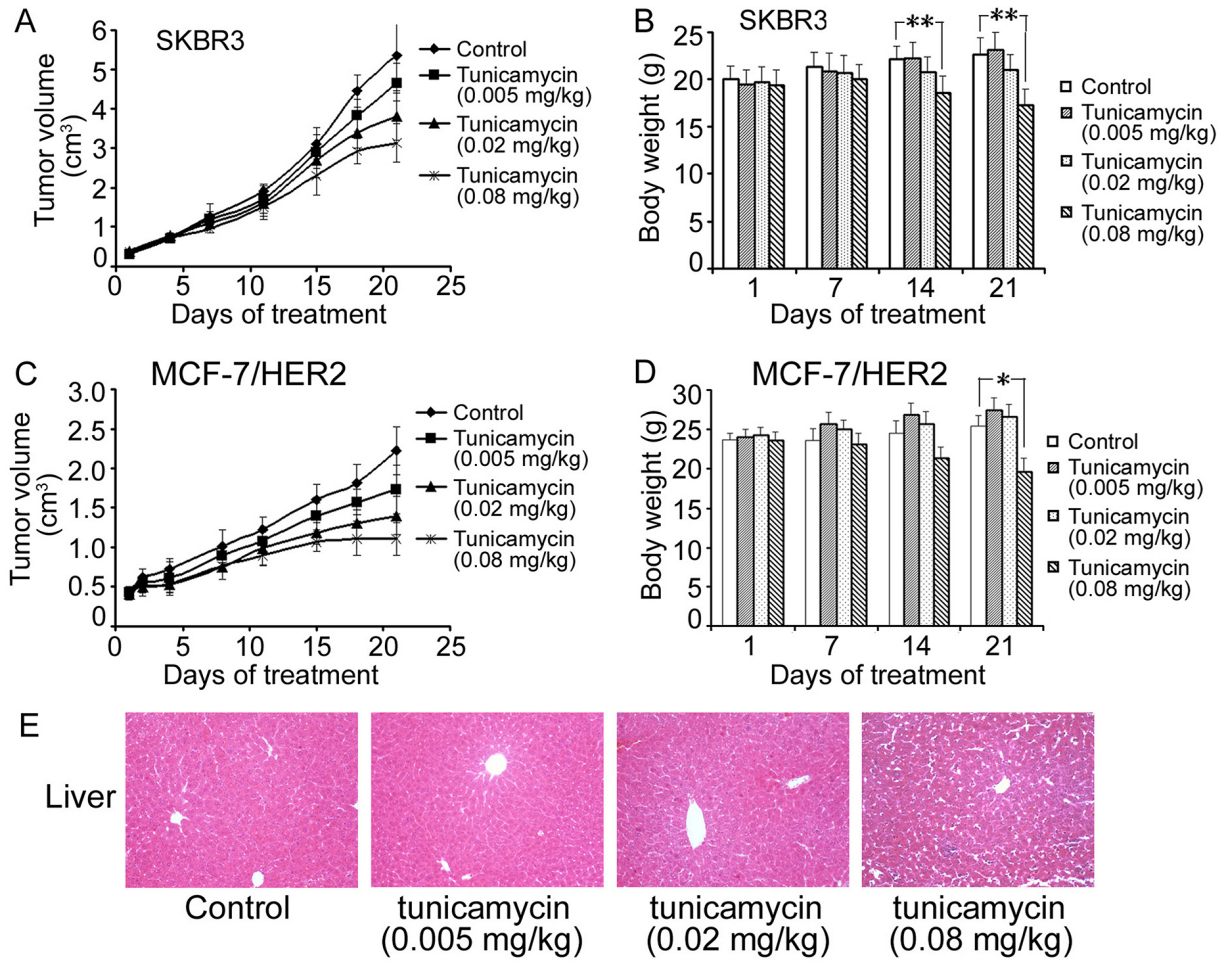
Antitumor effects of tunicamycin alone in xenograft model The nude mice bearing breast cancer cells SKBR3 **A, B.** and MCF-7/HER2 **C, D.** were treated as described in “Materials and Methods”. (A) and (C) Tumor growth curves during the treatment period by calculating the volume size of individual tumors. (B) and (D) Mean body weights for each group during the treatment period. **E.** HE staining of liver tissues in MCF-7/HER2-bearing mice treated with various doses of tunicamycin. Original magnification 200× and H&E staining. *P < 0.05, **P < 0.01.

Tunicamycin has broad, non-specific effects on all N-linked glycoproteins, so tunicamycin-induced N-glycosylation inhibition may produce systemic side effects. In order to explore a suitable dose of tunicamycin for treatment of tumor *in vivo*, we determined the antitumor activity of tunicamycin alone in nude mice bearing SKBR3 and MCF-7/HER2 cancer cells xenografts, respectively. Firstly, tunicamycin was injected intravenously twice a week for 3 weeks at the dose of 0.3 mg/kg, 0.6 mg/kg and 1.2 mg/kg in SKBR3 xenografts, respectively. However, all nude mice treated with tunicamycin died on days 17 for 0.3 mg/kg group, on days 15 for 0.6 mg/kg group and on days 11 for 1.2 mg/kg group, respectively. Tunicamycin-induced toxicity was dose-dependent; indicating that high dose of tunicamycin would cause systemic side effects. So we decreased the dose of tunicamycin to treat nude mice bearing SKBR3 xenografts again. Result showed that tunicamycin treatment at the dose of 0.005 mg/kg, 0.02 mg/kg and 0.08 mg/kg decreased tumor growth by 12.9%, 29.1% and 41.6% in SKBR3 xenografts, respectively (Figure 5A). We then repeated the effect of tunicamycin with the same doses on tumor growth in MCF-7/HER2 xenograft-bearing nude mice. As shown in Figure 5C, tunicamycin inhibited tumor growth by 21.9%, 32.5% and 50.4% in MCF-7/HER2 xenograft at the dose of 0.005 mg/kg, 0.02 mg/kg and 0.08 mg/kg, respectively. Tunicamycin treatment at the dose of 0.02 mg/kg and 0.005 mg/kg did not lead to any signs of abnormal behavior and significant weight loses in both xenograft mice bearing SKBR3 and MCF-7/HER2 cells (Figure 5B, 5D). However, the dose of 0.08 mg/kg tunicamycin caused dramatically weight loses in both xenograft mice. Histological analysis was also used to examine whether tunicamycin produced hepatocyte death. The results showed that the treatment of 0.08 mg/kg tunicamycin for 3 weeks resulted in lobular and portal inflammation in liver; however, hepatocytes were similar to normal when mice bearing MCF-7/HER2 were treated with tunicamycin at the dose of 0.005 and 0.02 mg/kg (Figure 5E), suggesting tunicamycin at 0.02 mg/kg was the optimal dose which did not result in significant side effects in mice.

**Figure 6:**
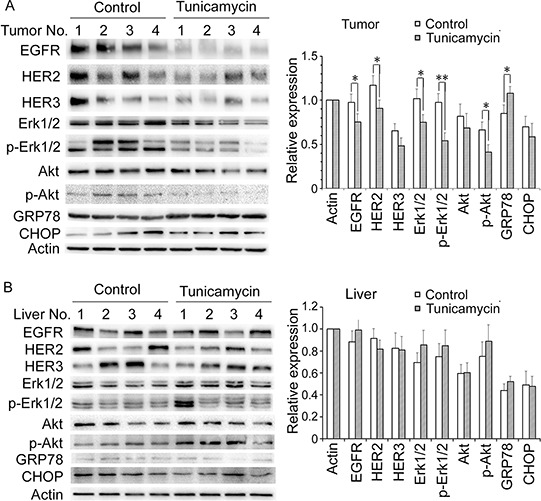
The effect of tunicamycin treatment at the dose of 0.02 mg/kg on protein expression in tumor A. and in liver samples B. in MCF-7/HER2 xenograft-bearing nude mice Four samples from each group with six mice were selected to do western blot analysis. Protein expressions were quantitated by densitometry and normalized against that of actin. Bars in the charts represent means ± SD of three independent experiments. *P < 0.05, **P < 0.01.

We then tested whether 0.02 mg/kg tunicamycin treatment could decrease the expression of EGFR family and induce ER stress in tumor and in liver tissues. Tumor and liver tissues were dissected from the MCF-7/HER2 xenograft treated with or without 0.02 mg/kg tunicamycin. Four samples from each group were selected for western blot analysis. Compared with control group, tunicamycin apparently disrupted the expression of EGFR, HER2 and HER3, as well as the MAPK and PI3K/Akt pathways in tumor samples (Figure 6A). GRP78 and CHOP, the hallmark of ER stress, were examined by western blot analysis. 0.02 mg/kg tunicamycin significantly increased GRP78 expression level in tumor; however, CHOP expression was not affected by tunicamycin treatment (Figure 6A). Notably, tunicamycin did not dramatically alter the expression of EGFR family and its downstream signaling pathway, as well as the GRP78 and CHOP level in liver tissue, demonstrating that tunicamycin at the dose of 0.02 mg/kg would not produce N-glycosylation inhibition and ER stress in normal tissue (Figure 6B).

**Figure 7:**
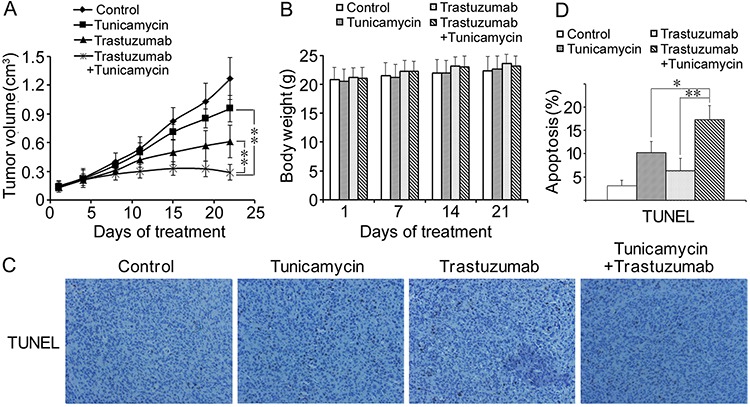
Antitumor effects of combined treatment of trastuzumab with tunicamycin in the nude mice xenograft model The nude mice bearing breast cancer cells MCF-7/HER2 were treated as described in “Materials and methods”. **A.** Tumor growth curves during the treatment period by calculating the volume size of individual tumors. **B.** Mean body weights for each group during the treatment period. **C.** Representative images of TUNEL staining of tumor sections. Brown coloration indicates apoptotic cells (original magnification 200 ×). **D.** The percentages of apoptotic cells were presented as average ± SD. *P < 0.05, **P < 0.01.

We then chose the dose of 0.02 mg/kg for next combined treatment in nude mice, because this dose can decrease the expression of EGFR family without causing any side effects on mice. Compared with the control group, the group of 0.02 mg/kg tunicamycin, 10 mg/kg trastuzumab and the combination treatment inhibited tumor growth by 24.6%, 50.7% and 77.2% in MCF-7/HER2 xenograft, respectively (Figure 7A). The combined therapy of tunicamycin with trastuzumab resulted in a significant reduction of tumor volume when compared with either tunicamycin or trastuzumab alone in nude mice bearing MCF-7/HER2 xenograft. The average weight of mice among the four groups was similar, demonstrating that all treatment were safe without producing toxicity (Figure 7B). TUNEL staining of tumor sections was performed to detect apoptosis *in vivo*. Apoptotic cells were found in all tumor sections from groups with tunicamycin, trastuzumab and the combined treatment, while few apoptotic cells were found in the control group. The percentage of apoptotic cells in the combined treatment group was significantly higher than those in the groups treated with tunicamycin or trastuzumab alone (Figure 7C and 7D).

## DISCUSSION

Trastuzumab has significantly improved outcome and remains the standard treatment in patients with HER2-overexpressing breast cancer; however, primary or acquired resistance to trastuzumab has been increasingly recognized as a major obstacle in the clinical management of this disease. Multiple and cross-talk pathways are involved in trastuzumab resistance which limit the clinical outcome. Multiple mechanisms may act simultaneously to confer resistance to the drug, so it is clinically unfeasible to extensively combine various therapeutic targets. However, it is a strategy to find out a multiple RTK inhibitor to increase the sensitivity of cancer cells to trastuzumab. Tunicamycin, an inhibitor of protein N-glycosylation, has been reported to abrogate RTK expression including EGFR family. In this study, we investigated whether tunicamycin could enhance the antitumor effects of trastuzumab against breast cancer *in vitro* and *in vivo*.

We firstly tested the growth inhibitory of tunicamycin alone in human breast cancer cells and in normal human cells. The results of SRB assay indicated that tunicamycin inhibited cell growth in a dose-dependent manner in breast cancer cells, as well as in normal human cells. No significant difference with IC_50_ values was found among breast cancer cells with low HER2-expressing cells MCF-7 and MDA-MB-231, and with high HER2-expressing cells MCF-7/HER2, SKBR3, MDA-MB-453 and BT-474 cells, demonstrating that the growth inhibitory effect of tunicamycin was not depended on HER2 expression levels. Similar growth inhibitory effects were observed in normal human cells as that in breast cancer cells, indicating that side-effects produced by tunicamycin should be considered when tunicamycin is used to treat cancer *in vivo*. The effects of combined treatment of tunicamycin with trastuzumab on cell growth were tested in breast cells with low or high HER2 expression. The results demonstrated that tunicamycin synergistically potentiated growth inhibition of trastuzumab in HER2-overexpressing breast cancer cells.

In agreement with previous reports, we also confirmed that tunicamycin or trastuzumab alone induced cells at G0/G1 arrest in HER2-overexpressing breast cancer cells. Compared with individual drug, the combined treatment of tunicamycin with trastuzumab enhanced G0/G1 arrest in MCF-7/HER2 cells and SKBR3 cells, but not in MCF-7 cells. Preclinical studies suggest that trastuzamab increases cell cycle arrest at G0/G1 phase via increasing the protein levels of p27, a cyclin dependent kinase inhibitor. Exogenous addition of p27 increased trastuzumab sensitivity [23]. Decreased p27 protein level has been associated with trastuzumab resistance and with poor prognosis in breast cancer cells [23]. In this study, tunicamycin or trastuzumab alone increased p27 protein level; however, the combined treatment of both drugs dramatically increased p27 expression as compared with individual treatment in HER2-overexpressing breast cancer cells. Cyclin D1 promotes the transition from G1 to S phase by binding to Cdk4. In this report, tunicamycin decreased cyclin D1 dose-dependently and trastuzumab did not resulted any changes of cyclin D1 expression; however, cyclin D1 expression levels were decreased obviously with the increasing concentrations of trastuzumab after combined treatment of trastuzumab with 1.0 μg/ml tunicamycin in HER2-overexpressing cells. These results demonstrated that tunicamycin increased trastuzumab-induced G0/G1 arrest through an increase of p27 expression and a decrease of cyclin D1 expression in MCF-7/HER2 and SKBR3 cells.

The most well-known effect of trastuzumab is the inhibition of the MAPK and PI3K/Akt pathways, which lead to an increase in cell cycle arrest, and the suppression of cell proliferation. The alternative elevations of other tyrosine kinase receptors and the activation of MAPK and PI3K/Akt pathways have been associated with trastuzumab resistance [11]. HER2-HER3 heterodimers is considered the most active EGFR family signalling dimer [24, 25]. However, trastuzumab has only a minor effect on ligand-induced HER2-HER3 dimerization [9, 10]. Previous reports have demonstrated that tuncamycin decreased EGFR expression through blocking EGFR maturation and its transportation from the ER to the cell membrane [17, 19]. In this study, the combined treatment of tunicamycin with trastuzumab significantly decreased EGFR, HER2 and HER3 protein and their phosphorylation levels compared with individual treatment in HER2-overexpressing cells MCF-7/HER2 and SKBR3. Tunicamycin also decreased the protein levels of Akt, Erk1/2 and their phosphorylation levels. The combination treatment greatly enhanced the decrease of phosphorylated Akt and phosphorylated Erk1/2, as well as Akt and Erk1/2 protein levels. These results indicated that combined treatment elevated the disruption of EGFR receptor family and its downstream pathways such as MAPK and PI3K/Akt, which improved the sensitivity of breast cancer cells to trastuzumab.

Tunicamycin inhibited biosynthetic process of protein N-glycosylation, which may cause ER stress and toxicity in normal tissues. In this report, in order to answer a fundamental question of whether a low dose of tunicamycin can disrupt N-glycosylation without toxicity to normal cells, the ER stress induced by tunicamycin was examined *in vitro* and *in vivo*. ER stress is modulated mainly by the ER chaperone GRP78, also known as binding immunoglobulin protein (BiP), which is involved in protein folding and in protein translocation [26]. CHOP, also called GADD153, is primarily pro-apoptotic and is one of the highly inducible genes during ER stress. Pro-survival GRP78 and pro-apoptotic CHOP are key opposing representatives of ER stress response [26]. In this report, both of GRP78 and CHOP protein levels were increased after treatment with tunicamycin in all tested cells including breast cancer cell lines and human normal cell lines (data not shown), indicating that tunicamycin-induced ER stress was not cell-type specific.

Many studies have reported the toxicity of tunicamycin *in vivo*. Cattle, sheep and pigs displayed clinical sensitivity to tunicamycin in the oral dose range of 0.5 to 1.0 mg/kg body weight; furthermore, a total cumulative oral dose of only 1 mg/kg was potentially lethal [27]. When mice were administered as a single intraperitoneal injection, the LD_50_ and LD_100_ of tunicamycin were 2.0 mg/kg and 3.5 mg/kg, respectively [28]. Some rats survived with daily subcutaneous injections of 50 μg/kg tunicamycin for up to 31 days [29]. Tunicamycin administered as a single subcutaneous dose of 200 μg/kg caused permanent destruction of seminiferous tubules in adult male rats [30]. Based on above data, nude mice involved in this study were firstly treated with tunicamycin at the doses of 0.3, 0.6 and 1.2 mg/kg body weight, respectively. However, all tunicamycin-treated mice died around two weeks. We then decreased the doses of tunicamycin in the following animal experiments. Nude mice bearing SKBR3 and MCF-7/HER2 xenograft were then treated with tunicamycin at the doses of 0.005 mg/kg, 0.02 mg/kg and 0.08 mg/kg, respectively. All doses inhibited tumor growth dose-dependently in both xenograft models; however, 0.08 mg/kg tunicamycin showed apparent totoxicity along with decreased weights. Compared with control group, 0.02 mg/kg tunicamycin inhibited tumor growth by 29.1% in SKBR3 xenograft and by 32.5% in MCF-7/HER2 xenograft without any signs of totoxicity. The mice treated with 0.005 mg/kg tunicamycin also showed no sign of abnormal behavior. A lots of papers indicated that tunicamycin induced ER stress in the liver in mouse [31–33]. Especially, Morin et al revealed that the major pathological manifestations of tunicamycin toxicity occurred first and foremost in the liver [28]. The results of histological analysis in this study showed that the treatment of 0.08 mg/kg tunicamycin resulted in lymphocyte accumulation in lobular and portal of liver; however, tunicamycin treatment at the dose of 0.005 and 0.02 mg/kg did not cause any changes in liver tissues. Above results indicated that 0.02 mg/kg tunicamycin was safe for treating mice bearing tumor xenograft.

We then examined the effects of 0.02 mg/kg tunicamycin on protein expressions such as GRP78, CHOP and EGFR family. Treatment with 0.02 mg/kg tunicamycin for 21 days resulted in significant decrease of EGFR, HER2 and HER3 in tumor samples from MCF-7/HER2 xenograft, demonstrating that the dose of 0.02 mg/kg tunicamycin abrogated the N-glycosylation process in tumor. Tunicamycin treatment dramatically elevated GRP78 protein; however, no alterations of CHOP protein levels were found in tumor tissues between treatment group and control group, indicating that tunicamycin induced ER stress in tumor tissues and that the ER stress was under the moderate levels since severe ER stress will lead to cell death through enhanced CHOP expression. By compared with control group, tunicamycin treatment at the dose of 0.02 mg/kg did not produce any changes of EGFR family and its downstream pathways, as well as GRP78 and CHOP protein levels in liver samples. This result indicated that the dose of 0.02 mg/kg tunicamycin was not enough to reach the point to inhibit N-glycosylation in liver tissue which proliferate slower greatly then tumor tissue.

Previous report showed tunicamycin at tolerable doses can reduce N-glycosylation in xenograft tumor and the activity of inhibiting N-glycosylation can sustain for up to 96 h after tunicamycin was intraperitoneally injected into nude mice bearing glioma cancer cells D54. Furthermore, D54 and U87MG glioma xenograft tumor experiments showed significant reductions in tumor growth following N-glycosylation inhibition and radiation therapy, consistent with an enhancement in tumor radiosensitivity [17]. Another study demonstrated that tunicamycin inhibits angiogenesis *in vivo* and reduces a double and a triple negative breast tumor growth in nude mice [21]. Due to disruption of EGFR family and no toxicity with the tunicamycin treatment at the dose 0.02 mg/kg, combined treatment of 0.02 mg/kg tunicamycin with trastuzumab was used to evaluate the antitumor growth effect in MCF-7/HER2 xenograft in this study. The result showed that 0.02 mg/kg tunicamycin was tolerable for nude mice and it enhanced the antitumor activity of trastuzumab by enhancing apoptosis. Above studies have provided evidence that low and tolerable dose of tunicamycin can be used to inhibit tumor growth and to sensitize tumor to trastuzumab treatment.

In this study, we confirmed that tunicamycin enhanced the antitumor activity of trastuzumab against HER2-overexpressing breast cancer through cell cycle arrest and apoptosis by increasing p21 expression and decreasing the EGFR family signaling pathways. We also proved that a tolerable dose of tunicamycin inhibited N-glycosylation and induced ER stress in tumor but not in liver tissues in xenograft-bearing nude mice. Our results raised the possibility that combinations of trastuzumab with tunicamycin or other N-glycosylation inhibitors may be a promising approach for improving the clinical activity of trastuzumab.

## MATERIALS AND METHODS

### Cell lines and cell culture

All human breast cancer cell lines used in this study were purchased from the Shanghai Cell Bank, the Institute of Cell Biology, China Academy of Sciences (Shanghai, China). All cell lines were maintained in a humidified atmosphere containing 5% CO_2_ at 37°C in different media supplemented with 10% FBS, 100 U/ml penicillin, and 100 μg/ml streptomycin. An immortalized human mammary epithelial cell line MCF10A was purchased from the American Type Culture Collection and cultured in DMEM supplemented with 5% horse serum, 20 ng/ml of epidermal growth factor, 0.5 μg/ml of hydrocortisone, 100 ng/ml of cholera toxin, 10 μg/ml of insulin, and penicillin/streptomycin. Breast cancer cell lines included MCF-7, MCF-7/HER2, MDA-MB-231, SKBR3, MDA-MB-453 and BT-474. MCF-7/HER2 was transfected MCF-7 cells stably overexpressing HER2 protein. MCF-7 cells were transfected with pcDNA3.1/HER2 plasmid and grew in media supplemented with G418 for several weeks. A single clone of stably transfected MCF-7/HER2 cell was selected as measured by western blot. Normal human cell lines included human embryonic kidney cells HEK-293T, immortalized human mammary epithelial cells HMLE, human umbilical vein endothelial cell HUVEC, and human hepatocyte-derived cells HL7702. All the cell lines were cultured in RPMI-1640 medium or DMEM medium.

### Sulforhodamine B (SRB) assay

Growth inhibition was determined using the SRB assay which estimates cell number indirectly by measuring total basic amino acids. Briefly, the cells were incubated in 96-well microtiter plates for 24 h. Following the addition of test drugs, the plates were incubated at 37°C for an additional 96 h in a 5% CO2 incubator. The culture medium was then discarded and the cells were fixed *in situ* by the gentle addition of 100 μl of cold 10% (w/v) trichloroacetic acid and incubated for 60 min at 4°C. The supernatant was discarded and the plates were washed five times with tap water and air dried. SRB solution (100 μl) at 0.4% (w/v) in 1% acetic acid was added and plates were incubated for 20 min at room temperature. After staining, unbound dye was removed by washing five times with 1% acetic acid and the plates were air dried. Bound stain was subsequently solubilised with 10 mM Tris (pH 10.5) and the absorbance was read at 515 nm on a Bio-Rad 550 ELISA microplate reader. Trastuzumab was obtained from Genentech/Roche, USA. Tunicamyicn was pursed from Sigma, USA.

### Drug interaction analysis

Drug interaction was determined by the isobologram and combination-index methods, derived from the median effect principle of Chou and Talalay using the CalcuSyn software [34]. Using data from the growth inhibitory experiments and computerized software, a combination index (CI) value is generated over a range of Fa levels from 0.05–0.95 (5%-95% growth inhibition). CI < 1, CI = 1, and CI > 1 indicate synergism, additive and antagonism, respectively.

### Cell cycle analysis by flow cytometry

The cells were trypsinized, washed in ice-cold 70% ethanol, and then stored at −20°C. Prior to analysis, the samples were washed twice in phosphate-buffered saline (PBS) and resuspended in a solution of propidium iodide (50 mg/ml) and RNase A (0.5 mg/ml) in PBS for 30 min in the dark. Data collected from each 10,000-cell sample was analyzed by Flow cytometry (Becton-Dickinson Co., USA).

### Annexin V-FITC/Propidium iodide assay

Annexin V-FITC/Propidium iodide (PI) binding assay was employed to determine the viable, early apoptotic cells. Following the recommended protocols of the Annexin V-FITC kit (BD Pharmingen, USA), the cells were seeded at 4 × 10^5^ cells/ml per well in 6-well plates. After treatment with tunicamycin, trastuzumab alone, or both, the cells were harvested and washed twice with ice-cold PBS and resuspended in 100 μl of binding buffer. A total of 5 μl of Annexin V-FITC and 10 μl of PI were added, and the mixture was incubated for 30 min in the dark. Finally, 400 μl of binding buffer was added to the cells, and the mixture was analyzed with a flow cytometer.

### Western blot analysis

The cells were trypsinized, washed with PBS, and then lysed with buffer containing 50 mM Tris-HCl (pH 7.5), 150 mM NaCl, 2 mM EDTA, 2 mM EGTA, 1 mM dithiothreitol, 1% Nonidet P-40, 0.1% SDS, protease inhibitors (1 mM PMSF, 5 mg/ml aprotinin, 5 mg/ml leupeptin and 5 mg/ml pepstatin), and phosphatase inhibitors (20 mM β-glycerophosphate, 50 mM NaF, and 1 mM Na_3_VO_4_). The tumor and liver tissue were washed with PBS and then were ground with the same lysis buffer. The lysates were incubated at 4°C for 20 min and centrifuged at 12,000 × g for 15 min. Equal amounts of lysate (20 μg or 30 μg) were resolved by sodium dodecyl sulfate polyacrylamide gel electrophoresis (SDS-PAGE) and transferred to polyvinylidene difluoride membrane (Millipore, USA). The membranes were blocked in 5% non-fat skim milk/TBST [20 mM Tris-HCl (pH 7.4), 150 mM NaCl, and 0.1% Tween-20] at room temperature for 2 h and detected with primary antibodies at room temperature for 2 h. The membranes were then blotted for 1 h at room temperature with an appropriate horseradish peroxidase-linked secondary antibody, followed by enhanced chemiluminescence Western blot detection reagents (Amersham Pharmacia Biotech, USA). The primary antibody cyclin D1, p21, cleaved PARP-1, GRP78 (N-20), actin, and the secondary antibody were purchased from Santa Cruz Biotechnology (USA); all the other primary antibodies were purchased from Cell Signaling Technology.

### Inhibition of tumor growth *in vivo*

The research protocol was inaccordance with the institutional guidelines of the Animal Care and Use Committee of Shandong University. The animals were housed under pathogen-free conditions. Female BALB/c (nu/nu) mice (20 ± 2 g, 4–6 weeks old) were purchased from the Animal Center of the China Academy of Medical Sciences (Beijing, China). The breast cancer SKBR3 or MCF-7/HER2 cells, 5.0 × 10^6^, suspended in 100 μl PBS, were subcutaneously inoculated into the lower right flank of the nude mice. Nude mice bearing MCF-7/HER2 xenograft were also implanted with 17β-estradiol tablets. When the tumors were 100–150 mm^3^, the mice were divided randomly into different groups (*n* = 6 in each group). The control group received PBS only. The mice receiving individual treatment of tunicamycin or the combined treatment of tunicamycin with 10 mg/kg trastuzumab were injected intraperitoneally with 100 μl drugs twice a week. The mice were treated for 3 weeks. The diameter of the tumor was measured twice a week with a caliper. Tumor volume was calculated with the following formula: *v* = ab^2^/2, where a and b are the long diameter and the perpendicular short diameter of the tumor, respectively. The body weights were measured once a week.

### Histological examination of liver tissues

Liver tissues obtained from nude mice were fixed with 10% formalin and then embedded in paraffin. Then 5 μm sections were cut and stained with hematoxylin-eosin (HE) staining.

### TUNEL staining

The apoptosis of paraffin-embedded tumor sections was detected using a TUNEL assay kit according to the manufacturer's manual (Roche). In brief, fixed and paraffin-embedded sections were dewaxed then permeabilized with proteinase K for 15 min at room temperature. Sections were treated with 3% H_2_O_2_ to block endogenous peroxidases and then incubated with equilibration buffer and terminal deoxynucleotidyl transferase (TdT) enzyme. Finally, sections were incubated with antidigoxigenin-peroxidase conjugate. Tissue peroxidase activity was evaluated through DAB application. Sections were examined under a light microscope.

### Statistical analysis

All quantitative data were subject to ANOVA to determine if there were significant differences between groups. For data groups satisfying the ANOVA criteria (*P* < 0.05), individual comparisons were conducted using the Student's *t*-test; *P* < 0.05 was considered significant.
